# Learning to Let Go: A Cognitive-Behavioral Model of How Psychedelic Therapy Promotes Acceptance

**DOI:** 10.3389/fpsyt.2020.00005

**Published:** 2020-02-21

**Authors:** Max Wolff, Ricarda Evens, Lea J. Mertens, Michael Koslowski, Felix Betzler, Gerhard Gründer, Henrik Jungaberle

**Affiliations:** ^1^Faculty of Psychology, Technische Universität Dresden, Dresden, Germany; ^2^Department of Psychiatry and Psychotherapy, Technische Universität Dresden, Dresden, Germany; ^3^MIND Foundation, Berlin, Germany; ^4^Department of Psychiatry and Psychotherapy, Charité Universitätsmedizin Berlin, Berlin, Germany; ^5^Department of Molecular Neuroimaging, Central Institute of Mental Health, Medical Faculty Mannheim, University of Heidelberg, Mannheim, Germany

**Keywords:** psychedelic therapy, cognitive behavioral therapy, avoidance, acceptance, psilocybin, lysergic acid diethylamide, ayahuasca

## Abstract

The efficacy of psychedelic-assisted therapies for mental disorders has been attributed to the lasting change from experiential avoidance to acceptance that these treatments appear to facilitate. This article presents a conceptual model that specifies potential psychological mechanisms underlying such change, and that shows substantial parallels between psychedelic therapy and cognitive behavioral therapy: We propose that in the carefully controlled context of psychedelic therapy as applied in contemporary clinical research, psychedelic-induced belief relaxation can increase motivation for acceptance *via* operant conditioning, thus engendering episodes of relatively avoidance-free exposure to greatly intensified private events. Under these unique learning conditions, relaxed avoidance-related beliefs can be exposed to corrective information and become revised accordingly, which may explain long-term increases in acceptance and corresponding reductions in psychopathology. Open research questions and implications for clinical practice are discussed.

## Introduction

In recent years, several early-phase clinical trials have provided evidence that classic serotonergic psychedelics—in most cases psilocybin, but also lysergic acid diethylamide (LSD) and the dimethyltryptamine (DMT)-containing brew ayahuasca—may occasion substantial and often sustained symptom reductions in patients treated for depression (1–3), psychological distress related to life-threatening illness (4–8), obsessive-compulsive disorder (9), and substance use disorders (10, 12). It has been proposed that psychedelic therapy works by reducing patterns of *experiential avoidance* and promoting more adaptive *acceptance* [(13); see below for definitions of these terms]. However, it remains largely unclear how psychedelic therapy may produce such change. Taking the perspective of cognitive behavioral therapy (CBT), and building on the recently proposed relaxed-beliefs account of psychedelics' acute brain action (14), the present article aims to clarify the psychological mechanisms underlying the acceptance-promoting effects of psychedelic therapy. We propose a conceptual model describing how psychedelic-induced belief relaxation, when combined with specific context factors that are typically present in psychedelic therapy, can facilitate the same acceptance-promoting learning process as that targeted by CBT interventions. In the following, we introduce the concepts of avoidance and acceptance, outline how CBT aims to promote acceptance, and review evidence that psychedelic therapy also promotes acceptance. We then briefly introduce the relaxed-beliefs account and, based on this, present our conceptual model of how psychedelic therapy promotes acceptance. This is followed by a discussion of open research questions and implications for clinical practice.

### Promoting Acceptance in Cognitive Behavioral Therapy

Many symptoms of mental disorders can be interpreted in terms of avoidance. This is most obvious in anxiety disorders, where avoidance of anxiety-provoking situations is a cardinal symptom, but it is also the case for many other diagnostic categories (15, 16): In depression, passivity, withdrawal, and rumination may serve to avoid unwelcome emotional experiences (17–20). In substance use disorders, intoxication may serve a similar purpose (21). In obsessive-compulsive disorder, washing rituals may neutralize worries about contamination (22), etc. When viewed as avoidance strategies, all these behaviors “work” in the sense that they diminish the threat of aversive experiences in the very short run. However, this small benefit comes at the immense longer-term cost of constraining the individual's personal liberty and perpetuating the disorder.

While the relevance of avoidance in psychopathology is recognized by all major schools of psychotherapy (23), it is especially emphasized in the so-called third wave of CBT. Here, experiential avoidance—defined as the attempt to evade, escape, or otherwise alter *private events* (i.e., emotions, thoughts, memories, body sensations, etc.) despite harmful long-term consequences—is considered a central factor underlying the development and maintenance of a wide range of psychopathologies (23, 24). Acceptance refers to the converse ability to allow private events to unfold without attempting to control them. Acceptance thus relates closely to the concept of mindfulness (25) and is considered a core mechanism of positive behavior change in third-wave CBTs such as dialectical behavior therapy [DBT; (26)], mindfulness-based cognitive therapy [MBCT; (27)], and acceptance and commitment therapy [ACT; (28)]. Beyond these “acceptance-based” approaches, CBT emphasizes the role of avoidance in anxiety disorders, but seeks to reduce harmful behaviors, including maladaptive patterns of avoidance, across diagnostic boundaries.

**Figure 1:**
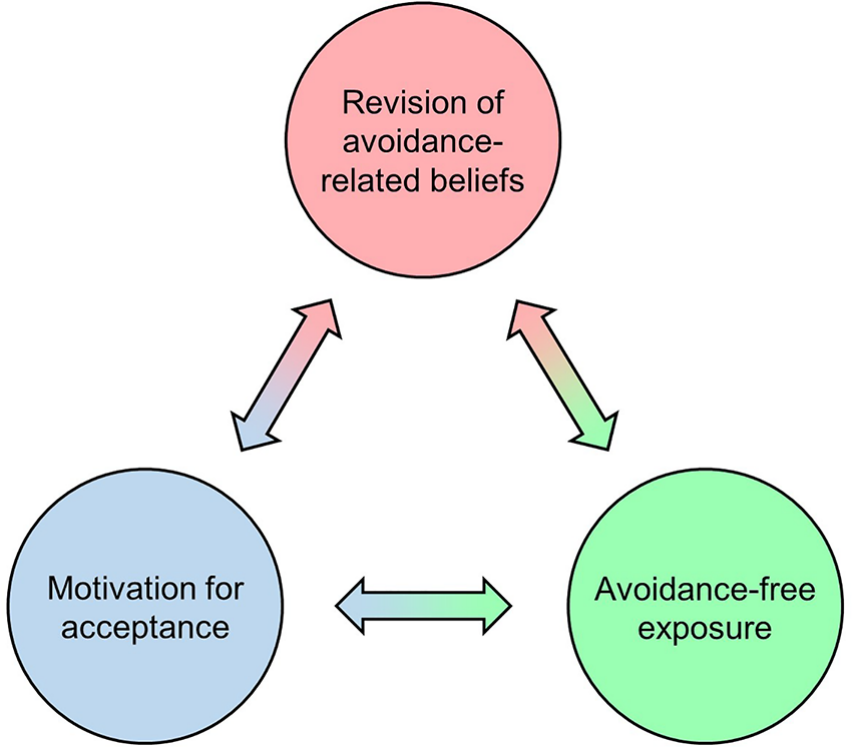
Interdependent cognitive, behavioral, and motivational aspects of an acceptance-promoting learning process. CBT aims to facilitate this learning process in order to promote lasting change from experiential avoidance to acceptance.

To facilitate lasting change from experiential avoidance to acceptance, cognitive-behavioral therapists use interventions aimed at different interdependent aspects of an acceptance-promoting learning process (see **Figure 1**). On a cognitive level, CBT seeks to enable the revision of avoidance-related beliefs, i.e., belief structures that motivate (and are sustained by) experiential avoidance. These may involve rather implicit negative expectancies (29) as well as preconscious assumptions and more explicit convictions about private events (e.g., “Anxiety is dangerous”), related self-conceptualizations (e.g., “I cannot handle anxiety”), and corresponding rules (e.g., “I must avoid anxiety at all costs”). Verbal interventions aimed at facilitating the revision of such beliefs can focus on changing either their content or functional impact on behavior, and may involve disputation *via* Socratic dialogue (30), metaphors (31), decentering or psychological distancing (32), defusion exercises (31), etc.

On a behavioral level, avoidance-free exposure is applied to induce corrective experiences with otherwise avoided private events. A prototypical case of exposure treatment is applied in classical CBT of anxiety disorders, which aims to reduce conditioned fear *via* extinction learning, i.e., by repeatedly confronting the patient with fear-provoking stimuli in the absence of aversive outcomes (33). Exposure in the form of behavioral experiments, i.e., gentle confrontation with avoided experiences to revise avoidance-related beliefs, is also applied beyond anxiety disorders [e.g., in depression; (34)]. Acceptance-based CBTs commonly pursue exposure through mindfulness-based exercises, which resemble classical exposure treatment of anxiety disorders in that a stimulus (in this case private events such as emotions, thoughts, memories, or body sensations) is openly attended to while desisting from avoidant responses (25, 27, 35). The similarity between mindfulness and other exposure treatments is reflected in that regular mindfulness exercise structurally and functionally affects the same network of brain regions that is also assumed to support fear extinction (36, 37), suggesting that this type of “internal” exposure can reduce avoidance *via* the extinction of threat responses to private events. Note that these events may still be unpleasant or painful even when they are no longer experienced as threatening. After all, acceptance-based CBTs do not primarily aim to change the form or frequency of aversive experiences, but to reduce harmful patterns of experiential avoidance (38).

On a motivational level, exposure is typically impeded by the fact that avoidant responses have been conditioned through reinforcement learning: As illustrated by the introductory examples above, avoidance often leads to immediate reductions in aversion. This negative reinforcement (i.e., removing an aversive stimulus or preventing an aversive event from happening) strengthens the avoidant response, meaning it will subsequently tend to occur with higher frequency, longer duration, greater magnitude, and/or shorter latency. By contrast, negative consequences of avoidance typically unfold much more slowly, and thus have little impact on operant learning. CBT seeks to counteract conditioned avoidance by increasing the patient's readiness to engage with aversive experiences (39), i.e., by building motivation for acceptance. This can be done by promoting insight into the longer-term costs of avoidance agendas (40), particularly with respect to their incompatibility with personally valued goals (38), and may involve motivational interviewing techniques (41). Likewise, avoidance motivation can be reduced through metaphors and experiential methods that demonstrate negative consequences or the futility of avoidance (38).

### Avoidance and Acceptance in Psychedelic Therapy

Psychedelic therapy refers to treatments for mental disorders where the patient is administered between one and a few moderate or high doses of a classic serotonergic psychedelic (psilocybin, LSD, or ayahuasca) under carefully controlled conditions in a professional clinical setting (42). During dosing sessions, which are embedded in a brief intervention model with preparatory and integrative counseling sessions, therapists usually take a non-directive approach. The patient, who is encouraged to turn attention inward, is mostly lying down, wearing eyeshades, and listening to a carefully selected playlist of music over headphones as the acute psychedelic experience unfolds [for concise summaries of the phenomenology of psychedelic states see (42, 43)].

There is mounting evidence that the positive long-term effects of psychedelic therapy are mediated by the quality of the acute psychedelic experience (44–47). Qualitative interviews with patients have shown that avoidance and acceptance are often central themes of their psychedelic experiences (13, 48–51), and patients commonly report transient episodes of struggle with intense aversion. These *challenging experiences*^1^ (42, 52, 53) are often characterized by extreme fear or panic, and can involve frightening imagery, unsettling body sensations, and the apprehension of immediate threat. This is the case even though patients are usually aware of their physical safety and the transitory nature of the experience. Attempts to exert control over challenging experiences (i.e., experiential avoidance) typically fail to bring the intended relief. Instead, patients frequently report that the experience only—and often immediately—assumed a more positive character when they eventually “surrendered” or “let go”, i.e., when they adopted an accepting attitude. The associated experience of an *emotional breakthrough* is commonly described as insightful and rewarding, and has been proposed to constitute a key component of psychedelic therapy (13, 54). Patients often experience episodes of unique openness to greatly intensified emotions during dosing sessions, and commonly describe the sensation that previously “hidden” or “suppressed” feelings became “accessible” or were “released” (13, 48, 49). Many patients report increases in emotional openness that last long after acute drug effects subside (13), and symptom reductions after psychedelic therapy are associated with enhanced neural measures of emotional responsiveness (55, 56). This is in line with quantitative evidence for lasting psychedelic-induced increases in the personality trait openness to experience (a negative correlate of experiential avoidance; 57) observed in clinical (58) and non-clinical samples (59–61). Psychedelic therapy thus appears to promote lasting change from experiential avoidance to acceptance (13). It has been proposed that this effect is causally related to the mentioned emotional breakthrough experiences, and a recent survey study lends preliminary support to this view (54). However, the underlying psychological processes have not been specified so far. Further below, we will present a conceptual model according to which psychedelic therapy can facilitate the same acceptance-promoting learning process as that targeted by CBT interventions (**Figure 1**). We base this argument on the recently proposed relaxed-beliefs account of the acute brain action of psychedelics (14).

### The Relaxed-Beliefs Account of Psychedelics’ Acute Brain Action

Carhart-Harris and Friston (14) proposed a unified account of the acute brain action of psychedelics. Although this recent theory still requires further empirical support, it widely accommodates the current state of knowledge about these substances' psychopharmacology, and parsimoniously explains their various psychotropic effects as the result of psychedelic-induced belief relaxation. The theory's neurobiological and information theoretical details are beyond the scope of this article, but understanding belief relaxation sufficiently to follow our argument requires a basic concept of *predictive processing*, arguably the leading unified account of brain and mind function (62, 63). According to the predictive processing framework, the brain with its hierarchical architecture entertains a hierarchically organized generative model of the current and general state of the world. At lower levels in the hierarchy, this model comprises rather momentary hypotheses about the causes of current sensory inputs (e.g., the perceptual belief that one is looking at a tree). At higher levels, the model becomes increasingly abstract, and forms more enduring hypotheses about the general nature of the world. At the highest levels, far removed from the sensorium, these beliefs (which do not need to be consciously held) are usually highly stable, such as the belief that a self exists and has certain properties.

To fulfill its biological function and control adaptive behavior in a complex changing environment, the brain needs the ability to form new beliefs and change existing ones. This ongoing process of belief updating is assumed to be guided by the principle of prediction error minimization: At each level of the hierarchy, probabilistic top-down predictions based on current beliefs are continuously compared with bottom-up inputs (basic sensory information at the lowest levels), and beliefs are adjusted in such a way that prediction errors (mismatches between predictions and inputs) are minimized. This process underlies the flexibility of the generative model, and ensures its correspondence with the external world. However, the sensitivity of beliefs toward ascending prediction errors may vary. Heavily-weighted (i.e., insensitive or “confident”) high-level beliefs are not easily updated, and often exert far-reaching constraining effects: They suppress prediction errors from certain lower-level parts of the model and keep them from impressing on higher levels. Thereby, these so-called compressive beliefs give the model stability and drastically reduce the number of its possible states, thus constraining phenomenal experience. For instance, the experience of seeing sounds (a case of visual-auditory synesthesia) should be largely prevented by heavily-weighted compressive beliefs along the lines of “sound is invisible” (the default state for non-synesthetes in normal waking consciousness).

The relaxed-beliefs account states that psychedelics acutely reduce the weight (i.e., confidence) of higher-level beliefs: By increasing their sensitivity toward prediction errors, otherwise stable beliefs become more easily updated. Furthermore, bottom-up information that is normally inhibited by compressive beliefs becomes liberated and is allowed to “travel up the hierarchy with greater latitude and compass” (14). This leads to a less constrained, more flexible state of mind which the authors refer to as the “anarchic brain”. A central characteristic of this state is increased context sensitivity, i.e., a heightened susceptibility toward ongoing processes in the internal and external context [or “set” and “setting”; see (64, 65)]. Processing domains which under normal circumstances are largely kept apart thus become more strongly interconnected. As a result, context-sensitivity phenomena like visual-auditory synesthesia (i.e., sensitivity of visual processes toward the auditory processing context, reflecting the relaxation of beliefs such as “sound is invisible”) are characteristic of psychedelic states. Beyond that, belief relaxation arguably accounts for the full spectrum of subjective phenomena associated with the psychedelic experience, including not only perceptual alterations but also visionary experiences, emotional lability, noetic insight, compromised sense of self, etc.

## A Cognitive-Behavioral Model of How Psychedelic Therapy Promotes Acceptance

**Figure 2:**
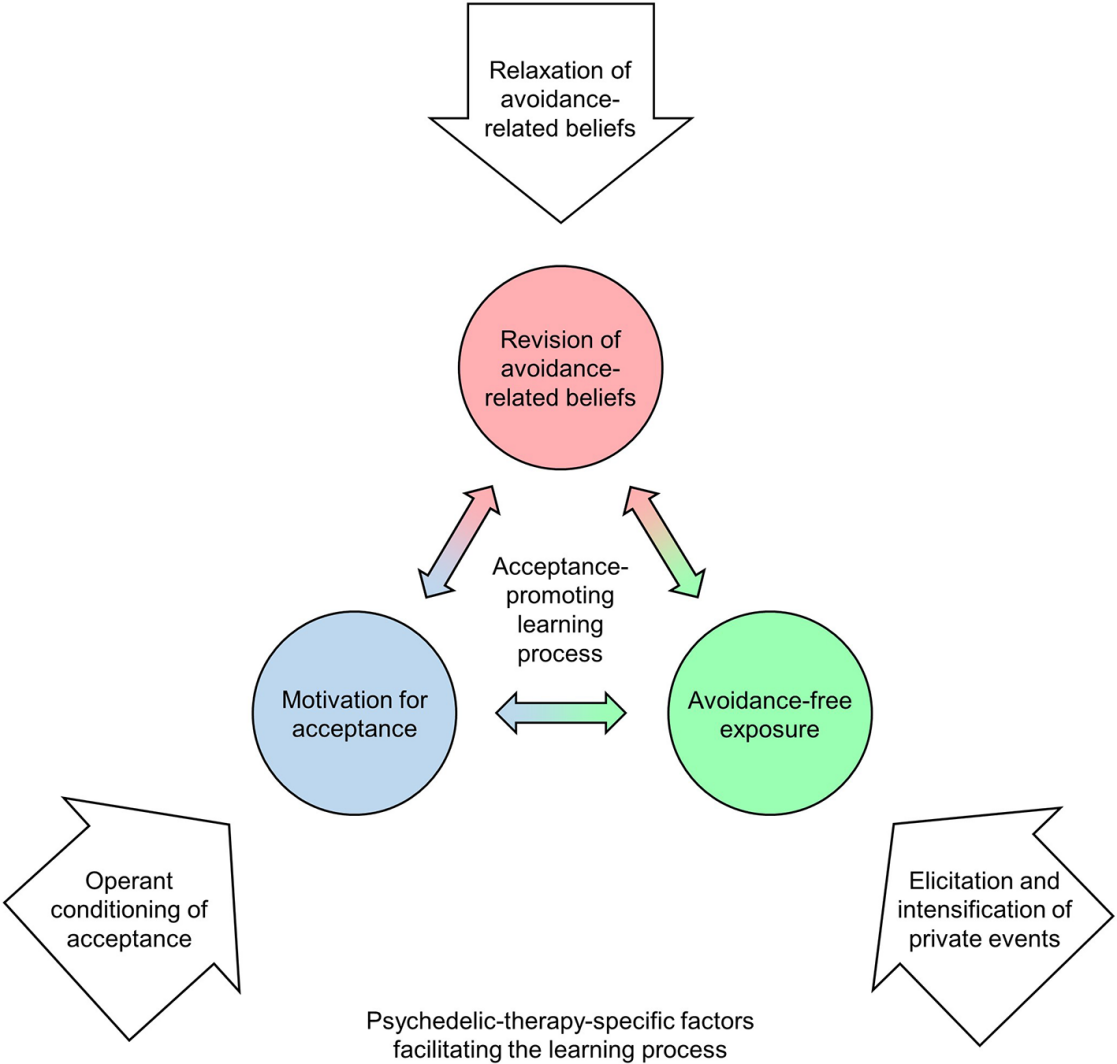
The proposed cognitive-behavioral model of how psychedelic therapy promotes acceptance. According to the model, psychedelic therapy facilitates the same learning process as that targeted by CBT interventions (see **Figure 1**). The proposed psychedelic-therapy-specific factors (white arrows) are assumed to arise from synergies between psychedelic-induced belief relaxation (14) and the particular context that is established according to psychedelic therapy protocols employed in contemporary research.

In this section, we describe some possible corollaries of belief relaxation that, in our view, can explain how psychedelic therapy promotes lasting change from experiential avoidance to acceptance: operant conditioning of acceptance, the elicitation and intensification of private events, and the relaxation of avoidance-related beliefs. According to our conceptual model (**Figure 2**), synergies between these psychedelic-therapy-specific factors can facilitate the same acceptance-promoting learning process as that targeted by CBT interventions.

### Operant Conditioning of Acceptance

A central cause of the stability of pathological avoidance is, as previously mentioned, that avoidant responses have often been repeatedly strengthened by negative reinforcement. It appears that this circumstance can be essentially reversed in psychedelic therapy, with the result that acceptance is conditioned instead of avoidance. Consider the following report of a psychedelic experience by a patient treated with psilocybin for depression:

*There was this huge terrifying creature with a rifle, and instead of running away, I looked at it, and it wasn't as scary as it had seemed. [My] fear subsided, it suddenly seemed ridiculous, I started laughing. If I had avoided it, it would have got more terrifying*.

Patient #4 (13)

Here, the patient's curious, accepting response to an aversive aspect of the experience (looking at the terrifying creature instead of running away) is negatively reinforced (the creature appearing less scary). Moreover, the patient has apparently somehow learned that an avoidant response (running away) would have been punished (the creature becoming even more terrifying). In what follows, we show that psychedelic-induced belief relaxation can account for such operant conditioning of acceptance.

#### Avoidance Sensitivity

As explained above, belief relaxation is thought to produce a relatively unconstrained state of mind characterized by increased sensitivity to context. This context sensitivity should emerge not only within perception (e.g., synesthesia between visual and auditory processes) but also between perceptual and affective-motivational processes. In the anarchic brain, increased bottom-up information flow from limbic into higher cortical areas (14) may allow avoidance-related processes to infiltrate and distort perception in ways that resemble synesthetic phenomena. Hence, avoidant states may bias perceptual belief updating towards what is (innately or by learning) associated with avoidance, leading to the emergence of threat-related perceptual content. For instance, the attempt to suppress a certain emotion may give rise to (more) unpleasant body sensations or repulsive imagery. The psychedelic state may thus involve a feedback loop whereby avoidant responses to aversive private events tend to increase aversion. We refer to this presumed circumstance as *avoidance sensitivity*, and propose that it constitutes a vital factor in psychedelic therapy.

Due to avoidance sensitivity, psychedelic states may be characterized by an intrinsic tendency to punish avoidance and reward acceptance. To prevent misunderstandings, this should not mean that avoidant behaviors always increase aversion in psychedelic states. For instance, physically escaping from a threatening external stimulus may in fact often be rewarded by decreased fear and feelings of relief (due to removal of the stimulus). We assume that punishment of avoidance *via* avoidance sensitivity is most likely to occur when avoidance is directed toward private events that are relatively unrelated to the immediate stimulus environment, i.e., in introspection as is encouraged in psychedelic therapy. Here, covert avoidance (e.g., trying to suppress aversive visual imagery by imagining something else) may produce more aversive content than it can eliminate. This is presumably intensified by additional context factors that are usually present in psychedelic therapy, where the patient is mostly lying down and wearing eyeshades. The resulting uncertain stimulus environment and associated deprivation from the grounding influence of well-defined sensory input (the notable exception being auditory stimulation with music, which is discussed below) can be assumed to strongly increase hallucinatory aspects of the psychedelic experience (66), and thus amplify avoidance sensitivity. This should be further enhanced by the patient’s lying-down body position, as reduced movement forbids many uses of active inference [i.e., acting on the environment to reduce uncertainty; (67)].

#### Shaping Acceptance

Given that avoidance sensitivity is presumably affected by the stimulus environment, the patient may use overt avoidance behaviors (removing the eyeshades, getting up and moving around, etc.) to seek distraction and tune down the intensity of aversive experiences. Such strategies, which can be actively supported by therapists, may in fact often reduce aversion to some degree. Nevertheless, due to encouragement by therapists and information provided in preparatory sessions, the patient may try and continue within introspection. Initial attempts at engaging with challenging experiences will likely reflect the patient's habitual patterns of responding, and may often rely on what has previously “worked” in everyday life: experiential avoidance. However, due to avoidance sensitivity, the attempt to exert control over the flow of events will likely aggravate aversive features of the experience, which may, in turn, elicit an even stronger avoidant response. Such escalation can be expected to proceed until the patient either resorts to overt avoidance or begins to desist from avoidance altogether. If neither occurs, the patient may soon find themselves in an intensely aversive state of panic^2^.

As soon as the patient spontaneously shows a minimum of acceptance toward an aversive aspect of the experience, this may initiate an operant process that can be described as an automatic form of shaping^3^. At first, the patient may only partially refrain from avoidance. Such a nuanced change in set may noticeably attenuate the emergence of threat-related perceptual content, thereby slightly reducing aversion. In the above example, as little as one curious glance at the terrifying creature (instead of, for instance, thinking about how to best run away from it) could already have made it appear significantly less frightening. Strengthened by such negative reinforcement, the initially only partial acceptance may subsequently generalize. Avoidance strategies are then increasingly let go of, and acceptance is brought to additional aspects of the experience. Here, broader acceptance can be assumed to yield stronger reinforcement. Under favorable conditions, this may allow the patient to rapidly achieve high levels of acceptance, even toward types of private events that are otherwise strongly avoided. The common phenomenon that a challenging psychedelic experience is suddenly resolved in a moment of breakthrough (54) could be explained as the result of such rapid shaping-like processes.

Certain additional context factors that are commonly present in psychedelic therapy (42) can be assumed to be crucial for the described process: The importance of assuming an accepting attitude toward the psychedelic experience is explicitly explained to the patient in preparatory sessions. The patient is instructed accordingly, and is encouraged to set an intention to “trust, let go, and be open” (70). Furthermore, therapists may serve as models for acceptance throughout the treatment, and may cue acceptance to the patient in dosing sessions. Patients have also attributed increases in acceptance of challenging psychedelic experiences to the encouraging influence of music (71). Not least, the purposefully created atmosphere of support, safety, and trust should be considered necessary for acceptance to be learned in psychedelic therapy.

### Elicitation and Intensification of Private Events

*Excursions into grief, loneliness and rage, abandonment. Once I went into the anger it went ‘pouf’ and evaporated*.

Patient #3 (13)

Such reports of exceptional openness to previously “hidden” or “suppressed” feelings during dosing sessions (13, 48, 49) suggest that conditioned acceptance may yield unique opportunities for exposure to private events that are otherwise avoided. Apart from the necessity to desist from avoidant responses, successful exposure treatment requires that suitable exposure targets (i.e., avoidance-related private events that are meaningfully related to the patient's psychopathology) are elicited and experienced with sufficient intensity. Hence, it appears advantageous that psychedelic-induced belief relaxation should involve the dissolution of top-down constraints on emotional, mnemonic, and perceptual processes (14). The resulting emotional effects, including the intensification of feelings, increased conscious access to emotions, and broadening of emotional range (43), may be of particular therapeutic value in this regard.

Considering that dosing sessions in psychedelic therapy usually last several hours, one might assume that the long duration alone ensures that therapeutically valuable exposure targets will sooner or later emerge. Furthermore, it is possible that the patient simply knows where in life avoidance is harming them [this could be further facilitated by the insight-promoting effects of belief relaxation; (14)], and actively engages with the respective topics. However, patients sometimes report a sense of being drawn into or guided towards “necessary” experiences, bearing the notion of an “inner therapist” (13), and suggesting that some highly efficient involuntary process of exposure target selection may be at work. It is an interesting possibility that such a process could be driven by periodic returns to avoidant responding (in behaviorist terms: *resurgences*): When an avoidant set is (re-)established for a brief moment, perceptual belief updating should be transiently biased towards what is associated with avoidance in the individual's memory. Thereby, periodic resurgences of avoidance may somewhat inevitably direct the flow of private events to what the patient most vigorously avoids in everyday life—which will likely relate to their individual psychopathology. Although speculative at present, it is conceivable that the surfacing of “forgotten” emotional memories [a regular occurrence in psychedelic therapy; (42)] and other phenomena that patients may attribute to an inner therapist would be facilitated by such a mechanism.

In the controlled context of psychedelic therapy, it can be expected that sensory deprivation in the visual, tactile, and proprioceptive domains will enhance the elicitation and intensification of private events. Another context factor of particular importance is music (72): Music increases psychedelic-induced visual imagery, which then often involves autobiographical memories (73), and can interact with self-referential processing in such a way that the personal meaningfulness of psychedelic experiences is increased (74). Perhaps most importantly, music’s powerful ability to evoke and amplify emotions is greatly enhanced in psychedelic states (71, 75, 76). Due to its central role in psychedelic therapy as a source of emotionality and meaning, music has been metaphorically referred to as “the hidden therapist” (72).

### Relaxation of Avoidance-Related Beliefs

Patterns of pathological avoidance are, as explained above, sustained by avoidance-related beliefs that motivate avoidant behavior and thereby impede corrective experiences. In terms of predictive processing, such rigid pathological beliefs are characterized by excessive weight (confidence), i.e., strong suppression of bottom-up information and insensitivity to prediction errors. In line with the notion that psychedelic therapy works by making rigid pathological belief systems malleable (14), we propose that the relaxation of avoidance-related beliefs opens a temporary window of plasticity through which these beliefs may undergo revision. However, this by itself should not warrant that avoidance-related beliefs are really changed, let alone with beneficial results. From a CBT perspective, positive results should be expected only when prediction errors encountered under belief relaxation are actually corrective with regard to dysfunctional beliefs. Following what has been said in the previous sections, this may in fact often be the case in psychedelic therapy: Enabled by operant conditioning of acceptance, relatively avoidance-free exposure to a multitude of greatly intensified private events should often produce experiences that strongly contradict negative expectancies. When the resulting large prediction errors impinge upon relaxed avoidance-related beliefs, they may exert a uniquely therapeutic corrective influence. Under favorable conditions, this could give rise to heavily-weighted and highly generalized *acceptance beliefs* (e.g. “Anxiety is not dangerous”). Apart from changes in explicit attitudes, belief relaxation may also facilitate the revision of more implicit expectancies, and reduce threat responses to private events through mechanisms related to extinction learning. In this respect, psychedelic therapy may resemble fear exposure treatment in CBT. Similar mechanisms have been proposed to underlie the therapeutic effects of mindfulness, which aims to broadly reduce reactivity to private events and is widely applied as a means of exposure in third-wave CBTs (25, 35–37). In line with the idea that psychedelic states can resemble the exposure-like quality of exercising mindfulness, psychedelics appear to enhance mindfulness capabilities (77–79), and mindfulness-related practices can enhance positive effects of psychedelics (80). It is well established that extinction learning in exposure treatments is most effective when negative expectancies regarding the outcomes of exposure are maximally violated (33). Psychedelic therapy appears to provide favorable conditions in this regard: First, the intense and often disturbing nature of the psychedelic experience may induce particularly negative expectancies about the outcomes of desisting from avoidance (e.g., “If I stop trying to control it, the anxiety will become absolutely unbearable”). By contrast, actual outcomes of avoidance-free exposure will often comprise a sense of breakthrough that is experienced as strongly rewarding, thus strongly violating negative expectancies. Following the relaxed-beliefs account, the effects of such expectancy violation on extinction learning should be further amplified by psychedelic-induced increases in sensitivity to prediction errors.

To summarize, psychedelic experiences that involve breakthrough experiences and episodes of relatively avoidance-free exposure to otherwise avoided private events may constitute unique learning conditions where relaxed avoidance-related beliefs can be revised with beneficial results. Corresponding changes in explicit attitudes, preconscious assumptions, and more implicit expectancies may profoundly transform the patient's way of relating to private events. The following patient report illustrates how these changes may lead to long-term increases in acceptance:

*I took away from the experience that I used to get angry about having anxiety, now I think I can have the anxiety, I can just feel it and it will go, I don't have to have the fear or run away*.

Patient #2 (13)

## Implications for Research

### Measuring Acceptance-Related Processes in Psychedelic Therapy

The proposed conceptual model (**Figure 2**) can be understood as a specific formulation of the more generic extra-pharmacological (EP) model of psychedelic drug action by Carhart-Harris and Nutt (44). At its core, the EP model assumes that long-term responses to psychedelics are predicted by relevant aspects of the acute drug response (which, in turn, results from interactions between drug-related, personal, and environmental factors). Applied to our model, long-term increases in acceptance and corresponding reductions in psychopathology should be especially pronounced following psychedelic experiences where operant processes engender episodes of relatively avoidance-free exposure to otherwise avoided private events, thereby enabling the revision of avoidance-related beliefs. Whereas qualitative analyses of patient interviews (13, 48, 49, 51) are compatible with this view, quantitative studies are needed to test and further develop the proposed model. This requires that relevant aspects of the acute psychedelic experience are adequately measured. To this end, we are currently developing a new questionnaire with separate scales for measuring the proposed acceptance-related processes in psychedelic states. To further clarify the role of acceptance as an underlying mechanism of change in psychedelic therapy, baseline and follow-up assessments in future clinical studies should include instruments for measuring experiential avoidance [e.g., (57, 81)] and related phenomena such as avoidant coping [e.g., (82)], thought suppression (83), and beliefs about the unacceptability of emotions (84). Assuming that acceptance is a central factor in psychedelic therapy, one should expect positive clinical outcomes such as symptom reductions to be at least partially mediated by decreases in experiential avoidance. Furthermore, research into the predictability of treatment outcomes based on pre-treatment avoidance levels could be an important basis for future clinical decisions (see our discussion of clinical targets below).

### Examining the Role of Challenging and Breakthrough Experiences

Challenging psychedelic experiences are potential starting points for acceptance-promoting learning processes, but are probably not always therapeutically valuable. In line with this, previous studies have found mixed results regarding long-term effects of challenging experiences: Roseman et al. (46) found that levels of anxiety and impaired cognition during psilocybin sessions predicted less positive clinical outcomes in depression patients. Likewise, a prospective survey study in a non-clinical sample (85) found that challenging psychedelic experiences had negative effects on subsequent well-being. Another survey (53) revealed that well-being was negatively related to the duration of challenging experiences, but positively related to their intensity. These seemingly contradictory results have been interpreted in the sense that “challenging experiences can indeed be therapeutically beneficial, but only if personal insight and/or emotional catharsis follows the relevant experience(s) of psychological struggle” (64). The same authors have recently developed a questionnaire for measuring this breakthrough quality of challenging experiences, and observed that emotional breakthrough predicted increases in well-being after naturalistic psychedelic use (54). We acknowledge that the intense relief inherent in such experiences may act as a massive reinforcement of acceptance. However, according to our tentative model, the therapeutic value of breakthrough experiences may lie not only in breakthrough itself but also in the preceding shaping of acceptance, subsequent exposure to otherwise avoided private events, and corresponding changes in avoidance-related beliefs. This distinction may be irrelevant in many cases, but it could be important in situations where the patient undergoes episodes of relatively avoidance-free exposure without previously having a challenging experience (and thus perhaps without experiencing a sense of breakthrough). This relates to the important question how the acute psychedelic experience and clinical outcomes are affected by a repetition of active dosing sessions. Modern clinical trials have involved between one and three active dosing sessions, but to date, no comparative studies have directly investigated the effects of repeated dosing on acute and long-term outcomes. From the learning perspective presented here, challenging experiences in a second or third dosing session might be reduced to the degree that previous sessions involved the revision of avoidance-related beliefs. However, the patient may still—or even more than in previous sessions—undergo episodes of therapeutically valuable exposure. Hence, to differentiate between the interrelated but distinct aspects of the proposed acceptance-promoting learning process, it should be attempted to assess these aspects separately and across repeated dosing sessions.

### Examining the Role of Ego-Dissolution Experiences

To date, most of the evidence supporting the EP model's core assumption that acute responses to psychedelics predict longer-term outcomes (44) relates to acute *ego-dissolution*, i.e., a transiently compromised experience of self that is characterized by a sense of unity with one's surroundings (86). From a predictive processing perspective, ego dissolution can be explained in terms of a transient disruption of self-related high-level beliefs (14, 87, 88). Blissful ego-dissolution and related phenomena such as “oceanic boundlessness” and “mystical-type experiences” have been shown to predict not only long-term increases in well-being (80, 85) and trait openness in non-clinical samples (59, 60, 89) but also positive clinical outcomes (5, 7, 11, 46). We propose the following interpretation of these findings: As discussed above, the patient may engage in overt avoidance behaviors (e.g., removing eyeshades or moving around) to reduce the intensity of acute drug effects, thereby reducing the likelihood of ego-dissolution. Likewise, covert (internal) avoidance strategies that involve self-referential processing (e.g., worrying) may to some extent impede the disruption of self-related high-level beliefs. By implication, ego-dissolution phenomena are less likely to occur when personal or contextual factors hinder acceptance-promoting learning processes such as that outlined in our conceptual model. Hence, the occurrence of mystical-type experiences or oceanic boundlessness can be seen as a (massively rewarding) consequence of having learned to let go of avoidance strategies [see (90) for recent evidence supporting this view]. The observation that blissful ego-dissolution is followed by long-term reductions in psychopathology, greater well-being, and increased openness may thus, at least in part, be explained in terms of reduced avoidance. In line with this idea, a recent survey study (91) found that the impact of acute mystical-type effects on decreases in depression and anxiety after naturalistic psychedelic use was entirely mediated by increases in psychological flexibility (a construct that is closely related to acceptance). Some positive effects of ego-dissolution could nonetheless be relatively unrelated to acceptance [e.g., see (92)]. To further investigate the therapeutic role of ego-dissolution experiences, future clinical studies should complement measures of ego-dissolution with measures of acceptance-related processes in the psychedelic state.

## Clinical Considerations

### Integrating Psychedelic Interventions Within Cognitive-Behavioral Treatment Models

According to the proposed model (**Figure 2**), psychedelics can facilitate the same acceptance-promoting learning process as that targeted by CBT interventions. This suggests that there are large potential synergies between CBT and psychedelic therapy. In line with this, it has been proposed that psychedelics could be fruitfully integrated within acceptance-based CBTs, most notably ACT [(13, 42, 93–96); for recent ACT-based protocols for psilocybin-assisted treatment of depression see (97, 98)]. We agree with this view, but emphasize that the proposed model is suited as a theoretical framework for integrating psychedelic therapy with not only ACT and other acceptance-based approaches but CBT more generally^4^. After all, all cognitive-behavioral treatment models seek to help patients find more adaptive (less avoidant) ways of relating to private events. Apparent disparities between third-wave and second-wave CBT models may be more accurately described as differences in viewing angles and preferred therapeutic techniques than differences in targeted psychological processes (99): Just as acceptance techniques used in ACT can be understood as methods for challenging avoidance-related beliefs, cognitive restructuring techniques in traditional CBT can be seen as ways of encouraging acceptance (100). From this perspective, it appears that limiting the integration between psychedelic therapy and CBT to techniques belonging to one or the other CBT model would unnecessarily narrow down the repertoire of available interventions. Hence, we propose an empirical approach to the question of which particular CBT interventions are best suited to amplify the acceptance-promoting effects of psychedelic therapy: Future clinical studies with psychedelics should investigate how effect sizes are affected by systematically varying psychological interventions, and assess whether these effects are moderated by patient characteristics. Such variations should not be restricted to preparatory and integration sessions, but may also involve gentle deviations from the prevailing traditional non-directive approach for dosing sessions (e.g., therapists actively addressing avoidance-related beliefs).

Whenever considering acceptance as a mechanism of positive change, it is important to note that acceptance should not be seen as an end in itself, but rather as a requirement for living in accordance with one's chosen values (38, 100). The reciprocal relationship between acceptance and values may be reflected in the observation that patients commonly report reconnecting with personal values or discovering new ones through the psychedelic experience (13, 48, 101, 102). On this basis, it can be assumed that treatment outcomes could be optimized by including values work in treatment. Psychedelic therapy protocols that involve values-based interventions have been described [e.g., (97, 103)]. To further improve treatment models, the impact of such interventions on treatment outcomes should be investigated systematically.

### Direct Implications of the Model for Clinical Practice

A central hypothesis presented here is that psychedelics can transiently compromise the effectiveness of avoidance strategies for (in the very short run) reducing aversive states. This may constitute a major difference between psychedelic therapy and more conventional methods in psychotherapy (where the patient can more easily reduce aversion by resorting to avoidance), and has important ethical implications for clinical practice. Most importantly, for the patient to be able to provide informed consent, they should be thoroughly informed about potential avoidance-impeding effects of the treatment. This requires that patients are given the opportunity to learn what avoidance is, and may involve not only educational but also experiential elements. Hence, the process of enabling valid informed consent for psychedelic interventions may already necessarily involve substantial elements of psychotherapy.

According to our model, operant conditioning of acceptance requires the patient to “start the ball rolling” by spontaneously showing a minimum of acceptance toward an aversive aspect of the experience at some point. Apart from the obvious implications that are already accommodated by current protocols for preparatory sessions (e.g., building an atmosphere of safety and trust; training mindfulness; setting intentions for acceptance), this may inform therapeutic strategies for dealing with challenging experiences: Whereas therapists may initially attempt to facilitate breakthrough by encouraging acceptance, challenging experiences that persist for longer periods of time may indicate that the patient cannot (at present) desist from avoidance sufficiently to induce shaping of acceptance. This situation entails the risk that motivation for acceptance is markedly decreased and further attempts are impeded. It may therefore in some situations be therapeutically beneficial to actually support the patient's decision for avoidant responding before encouraging acceptance again. The ability to gauge the individual patient's distress tolerance on a moment-to-moment basis and strike a sensible balance between encouraging acceptance and supporting avoidance can be considered a key requirement for psychedelic therapists, and should be trained accordingly. It can be argued that such perspective-taking requires first-hand experience with psychedelic states [see (104) for a discussion of this matter].

The proposed model explains increases in acceptance after psychedelic therapy in terms of revised avoidance-related beliefs. After the dosing session, newly established acceptance beliefs and corresponding behavior change may be more or less enduring depending on how generalized and heavily-weighted those beliefs are. In any case, long-term outcomes should be substantially affected by the learning conditions that the patient is exposed to after acute drug effects subside. In most cases, the patient will soon return to an environment that has been to some extent organized around avoidance goals. Continued psychotherapy may then help identify and change persistent habits, routines, and other circumstances that impede the pursuit of more acceptance-oriented approach goals. The same applies to individual deficits that hinder the abandonment of avoidant coping strategies (e.g., deficient social competencies or problem-solving abilities). Therapists should also pay attention to how the patient's social environment responds to changes in behavior and attitudes. For instance, returning to an emotionally invalidating or dismissive environment without appropriate therapeutic support may result in rapid re-establishment of pathological avoidance-related beliefs. It appears unlikely that two or three integration sessions suffice to address such challenges in all cases. Hence, the prevailing brief intervention models employed in contemporary psychedelic therapy studies (42) may not adequately serve the needs of all patients, particularly those with limited personal or social resources.

#### Clinical Targets

Assuming that promoting acceptance is one of its core mechanisms, psychedelic therapy can be expected to have most pronounced positive effects in those mental disorders that are typically characterized by excessive experiential avoidance. This encompasses many of the most prevalent mental disorders, including some that are already in the focus of psychedelic research (e.g., depression and addiction) and others for which modern clinical trials have not yet been conducted, such as panic disorder, posttraumatic stress disorder (PTSD), or psychosomatic disorders. Psychedelic therapy may hold less promise for conditions where avoidance is not considered a central factor, such as attention-deficit/hyperactivity disorder (ADHD) or psychotic disorders (15). Especially in the latter patient group, this may shift the risk-benefit ratio against psychedelic interventions. In line with this, pre-prohibition clinical studies, which tested psychedelics for mental disorders across the board, found positive results mostly in (then so-called) “psychoneurotic” disorders (105).

Within suitable diagnostic categories such as depression or addiction, how to determine if an individual patient is likely to benefit from acceptance-informed psychedelic therapy? On the one hand, it can be speculated that those patients who exhibit particularly high levels of experiential avoidance at baseline have the greatest potential for improvement. On the other hand, there may be a tipping point at which patterns of avoidance are too inflexible to make use of challenging psychedelic experiences. According to the proposed model, the shaping-like operant process of conditioning acceptance can be initiated only when the patient is able to show a minimum of acceptance spontaneously. If this is impossible due to personal (or contextual) factors, this may give rise to prolonged challenging experiences that have no therapeutic value or could even aggravate avoidance-related beliefs. One might assume that such tipping points are localized around the threshold where the inflexibility and pervasiveness of experiential avoidance and related patterns of emotion dysregulation justify the diagnosis of a personality disorder (e.g., avoidant personality disorder or borderline personality disorder). However, excluding patient populations based on such ideas seems premature without empirical support, especially when considering the substantial need to improve current treatments for personality disorders. Zeifman and Wagner (96) made a strong case for exploring the incorporation of psychedelics within interventions for borderline personality disorder (e.g., DBT), basing their argument partly on these substances' acceptance-promoting effects. Further research into the predictability of acute and long-term responses to psychedelics is needed to determine criteria for psychedelic treatment eligibility. While it is common practice in clinical trials to exclude patients based on rather trait-like attributes (e.g. diagnosis of a personality disorder), state measures (e.g. quality of the therapeutic relationship or clarity of acceptance-oriented intentions) may eventually emerge as more robust (and perhaps mediating) predictors of treatment outcomes.

### Applicability to MDMA-Assisted Psychotherapy

Although not a classic psychedelic, the entactogen 3, 4-methylenedioxymethamphetamine (MDMA) is applied in therapeutic interventions following protocols which closely resemble those used for psychedelic therapy (106). For some patients who are unsuited (or unwilling) to undergo treatment with classic psychedelics, MDMA may be considered as a more easily tolerable alternative (106). MDMA-assisted psychotherapy shows remarkable promise as a treatment for PTSD (107), and appears to work by facilitating engagement with traumatic memories and supporting fear extinction (108). Thus, as is proposed here for (classic-)psychedelic therapy, MDMA-assisted psychotherapy may parallel CBT in promoting motivation for acceptance, avoidance-free exposure, and the revision of avoidance-related beliefs. However, the mechanisms underlying these processes are likely different for MDMA and classic psychedelics given their distinct psychopharmacological action. Many of these differences, which cannot be discussed at length here, are potentially relevant for clinical decisions. Perhaps most importantly whereas we propose that classic psychedelics increase motivation for acceptance *via* avoidance sensitivity (making avoidance more aversive), MDMA seems to facilitate engagement with otherwise avoided private events primarily by attenuating the fear response (making acceptance less aversive). Clinical applications of MDMA-assisted psychotherapy are currently being extended beyond PTSD (106), and PTSD may become a target of treatments with classic psychedelics in the future (109). Hence, commonalities and differences in the psychological mechanisms underlying MDMA- and (classic-)psychedelic-assisted therapies may become important considerations in future clinical decision making, and should be investigated accordingly.

## Conclusion

The therapeutic effects of psychedelics appear to depend on psychological processes that are evoked by synergies between these substances' pharmacological action and the context in which they are administered. To better understand and further develop psychedelic therapy, theoretical models that specify these processes are needed. Here, we took a CBT perspective and proposed such a model based on Carhart-Harris and Friston's (14) relaxed-beliefs account of psychedelics' acute brain action: When combined with specific context factors that are typically present in psychedelic therapy, belief relaxation can increase motivation for acceptance *via* operant conditioning, thus engendering episodes of relatively avoidance-free exposure to greatly intensified private events. Under these unique learning conditions, relaxed avoidance-related beliefs can be exposed to corrective experiences and become revised accordingly, potentially leading to long-term increases in acceptance and associated reductions in psychopathology. This model shows substantial parallels between psychedelic therapy and CBT that may be harnessed by using CBT as a therapeutic framework for psychedelic interventions. Empirical research is needed to validate and further develop the proposed model and, more generally, to examine the relative importance of acceptance as a mechanism of action in psychedelic therapy. Therefore, appropriate instruments for measuring processes related to avoidance and acceptance in psychedelic states must be developed. Although still requiring further empirical support, the proposed model demonstrates the usefulness of the relaxed-beliefs account as a basis for building theories of the therapeutic effects of psychedelic drugs.

## Author Contributions

MW and HJ conceived the central theoretical ideas presented in this article. RE, LM, MK, FB, and GG provided critical feedback. The manuscript was written primarily by MW with contributions from RE, LM, MK, FB, GG, and HJ.

## Funding

Open Access Funding by the Publication Fund of TU Dresden.

## Conflict of Interest

The authors declare that the research was conducted in the absence of any commercial or financial relationships that could be construed as a potential conflict of interest.

## Footnotes

1 Contrasting the colloquial “bad trip”, this intentionally neutral term accommodates the possibility that these experiences may in fact, as discussed below, be therapeutically valuable.

2 The described process bears some resemblance to the escalation of anxiety in panic attacks, which is assumed to be driven by catastrophic misinterpretation of (and associated avoidant responses to) body sensations (68).

3 Shaping is a conditioning paradigm where the subject's spontaneous behavior is gradually changed towards a target behavior by differential reinforcement of successive approximations (69).

4 Beyond CBT, most other schools of psychotherapy also recognize the role of experiential avoidance or related concepts in human suffering (21). Therefore, although our model is formulated in CBT terms, it may still add a valuable perspective to how proponents of other schools (e.g., psychodynamic therapy) understand the therapeutic value of psychedelic states.
